# Correlation Analysis Between HLA Polymorphisms and Immune Response to Hepatitis B Vaccine in Children with Acute Lymphoblastic Leukemia

**DOI:** 10.3390/vaccines14020145

**Published:** 2026-01-30

**Authors:** Rui Zhang, Tian Yang, Yijin Gao, Hua Zhang, Yi Fei, Laibao Yang, Pengfei Deng

**Affiliations:** 1 Department of Immunology, Shanghai Pudong New Area Center for Disease Control and Prevention (Shanghai Pudong New Area Health Supervision Institute), Zhangyang Road 3039, Shanghai 200136, China; zhangrr692025@163.com (R.Z.); tyang@pdcdc.sh.cn (T.Y.); fy_yf2004@163.com (Y.F.); 2 Department of Hematology & Oncology, Shanghai Children’s Medical Center, School of Medicine, Shanghai Jiao Tong University, Shanghai 200127, China; gaoyijin@scmc.com.cn (Y.G.);

**Keywords:** HLA polymorphism, hepatitis B vaccine, acute lymphoblastic leukemia, pediatric immune response

## Abstract

Background: The human leukocyte antigen (HLA) is crucial for antigen presentation and vaccine efficacy. This study examined the association between HLA polymorphisms and the immune response to hepatitis B vaccination in children with acute lymphoblastic leukemia (ALL). Methods: 101 pediatric ALL patients at Shanghai Children’s Medical Center affiliated with Shanghai Jiaotong University School of Medicine who tested negative for hepatitis B surface antibody (anti-HBs) and were not infected with hepatitis B received three doses of the hepatitis B vaccine. Anti-HBs titers were measured before and after vaccination. Participants were divided into high- and low-response groups based on post-vaccination anti-HBs titers. Sequence-specific primer polymerase chain reaction (PCR-SSP) was used to genotype HLA-A, -B, -Cw, -DRB1, and -DQB1 alleles. Results: Pre-vaccination anti-HBs titers were 3.38 ± 2.97 mIU/mL, and the post-vaccination seroconversion rate was 100% with mean titers of 429.61 ± 303.13 mIU/mL (*p* < 0.001). Following immunization, the low-response group (11.88%) had an anti-HBs titer of 56.47 ± 28.38 mIU/mL, while the high-response group (88.12%) had an anti-HBs titer of 479.93 ± 287.70 mIU/mL. There were significant differences in allele frequencies of B*3501 and Cw*0303 between the two response groups (*p* < 0.05). Binary logistic regression analysis showed that the B*3501 allele was negatively correlated with the anti-HBs response level (*p* < 0.05). Conclusions: HLA-B*3501 may be associated with lower antibody response levels in children with ALL who completed the full hepatitis B vaccination series. All these children demonstrated protection against the hepatitis B virus (HBV). We will subsequently validate the association between HLA-B*3501 and the level of hepatitis B vaccine immune response in children with ALL through expanding the sample size or conducting a multicenter study.

## 1. Introduction

Based on the Global Burden of Disease database, from 1990 to 2021, the global incidence, mortality, and disability-adjusted life years (DALYs) of acute lymphoblastic leukemia (ALL) showed a downward trend. However, 53,485 new cases of childhood ALL were reported globally in 2021, which led to 23,991 deaths and 1,960,922 DALYs [1]. The data therefore demonstrates that the health burden of ALL on children remains substantial and cannot be neglected. The immunocompromised state of children with ALL, arising from both the pathophysiology of the disease and therapeutic measures, significantly heightens their vulnerability to infections compared to immunocompetent children [2,3].

Hepatitis B virus (HBV) infection persists as a significant global public health issue. It is estimated that approximately 296 million individuals are living with chronic HBV infection, and approximately 884,000 deaths each year are attributable to HBV-related diseases [4,5]. Recent research reveals that China shoulders the highest global burden of HBV infection, with 75 million chronic carriers, accounting for one-third of the infected population worldwide [6]. However, for pediatric patients with ALL, chemotherapy or hematopoietic stem cell transplantation may induce immunosuppression, thereby significantly increasing the risk of HBV infection or reactivation. Previous studies have established that HBV reactivation is a significant concern in patients with hematological malignancies. Among the broader group of patients who are hepatitis B surface antigen (HBsAg)-negative, the overall reactivation rate is approximately 3.1%, with patients suffering from ALL being at a particularly elevated risk [7]. It is crucial to recognize that the population of “HBsAg-negative” patients is heterogeneous and includes individuals with a past resolved hepatitis B infection (hepatitis B core antibody-positive, anti-HBc-positive). A prospective cohort study revealed that 39% of untreated HBsAg-negative pediatric ALL patients were anti-HBc-positive [8], and this subgroup accounts for the substantial majority of reactivation events, with a cumulative incidence reaching up to 10.5% within three years of chemotherapy [7]. In contrast, the risk of reactivation is extremely low for patients who are truly uninfected (HBsAg-negative and anti-HBc-negative); however, they remain susceptible to new HBV infection.

The hepatitis B vaccine is widely recognized as the most effective way to prevent HBV infection. It has been proven to reduce the risk of developing acute and chronic hepatitis B, cirrhosis, and hepatocellular carcinoma [9]. Currently, research on hepatitis B vaccines primarily focuses on antibody levels and healthy populations, with extremely limited studies conducted on ALL survivors who have no history of hepatitis B infection. The efficacy of the hepatitis B vaccine varies considerably among individuals, influenced by multiple factors. The major histocompatibility complex (MHC) haplotypes and genetic polymorphisms, which are closely associated with immune regulation, are key genetic factors that affect vaccine effectiveness [10,11]. The human leukocyte antigen (HLA) system, encoded by the MHC on chromosome 6, comprises three main classes: Class I, comprising HLA-A, -B, and -C, is expressed on nucleated cells; Class II, including HLA-DR, -DQ, and -DP, is present on antigen-presenting cells; Class III encompasses immunoregulatory components [12]. HLA Class II molecules play a crucial role in mediating vaccine responsiveness by presenting HBsAg to CD4+ T lymphocytes, thus initiating humoral immune responses. Current evidence suggests that DPB1*04:01 and DPB1*04:02 enhance hepatitis B surface antibody (anti-HBs) seroconversion, while DPB1*05:01 has an inhibitory effect [13]. Specifically, DRB1*14:54 may reduce anti-HBe responsiveness due to impaired HBeAg peptide binding [14], and DQB1*03 and DQB1*05 alleles are associated with persistent HBV infection [15]. Population-based analyses indicate that HLA-DR/DQ haplotypes can account for up to 10% of the variance in vaccine response within the African population [16].

Although extensive research has confirmed an association between HLA polymorphisms and hepatitis B vaccine immunogenicity, there remains a lack of in-depth genetic exploration within the ALL population. Particularly in China, the large size of the ALL population, low vaccination rates, and insufficient awareness and parental initiative regarding vaccination have led to a scarcity of relevant studies. Therefore, this study will explore the potential influence of HLA polymorphism on the antibody response levels following hepatitis B vaccination in pediatric ALL patients.

## 2. Materials and Methods

### 2.1. Study Design and Objectives

This prospective study recruited 101 children diagnosed with ALL at Shanghai Children’s Medical Center, affiliated with Shanghai Jiaotong University School of Medicine, from 2021 to 2024. Before their inclusion in the study, written informed consent was procured from all legal guardians. Recombinant hepatitis B vaccine (01190526A, Hissen Vaccine, Dalian, China) was used for the study subjects and administered as 3 doses of 10 μg at 0, 1, and 6 months. Venous blood specimens were collected at two discrete time points: (1) before vaccination, which served as the baseline, and (2) one month after completion of the hepatitis B vaccination program.

Genomic deoxyribonucleic acid (DNA) was extracted from peripheral blood samples. Anti-HBs titers were quantitatively determined via an enzyme-linked immunosorbent assay (anti-HBs ELISA kit from the Shanghai Zheke Biological Engineering Center, Shanghai, China) in conjunction with the EVO automatic enzyme immunoassay system (Tecan Group Ltd., Zürich, Switzerland). The detection limit was >0 mIU/mL, and the quantification range of the anti-HBs ELISA assay was 0–1000 mIU/mL.

China’s Technical Guide for Adult Hepatitis B Immunization defines anti-HBs levels ≥ 10 mIU/mL as protective. In contrast, Public Health England and recent studies suggest that ≥100 mIU/mL may also serve as an alternative threshold [17,18,19]. Therefore, employing 10 mIU/mL and 100 mIU/mL as thresholds, individuals with anti-HBs levels below 10 mIU/mL after hepatitis B vaccination were deemed to be in a seronegative state and were incorporated into the non-response group. Individuals with anti-HBs levels ≥ 10 mIU/mL are associated with complete clinical protection, yet they may experience reduced durability and are thus included in the low-response group. Conversely, individuals with anti-HBs levels equal to or exceeding 100 mIU/mL were classified as complete vaccine responders and included in the high-response group.

### 2.2. Inclusion and Exclusion Criteria

Inclusion Criteria: (1) Children who have been clinically diagnosed with ALL. (2) Children aged 0 to 18 years with a history of complete hepatitis B vaccination. (3) Children tested negative for HBV serological markers, defined as HBsAg negative, anti-HBc negative, and anti-HBs titer < 10 mIU/mL, to ensure study subjects were genuine HBV susceptible individuals without prior infection or occult infection. (4) Children who have not used immunosuppressants or received blood transfusions within the past 3 months. (5) Children must be in a state of immune restoration with normal immune function for at least 12 months after chemotherapy, with a blood test indicating a neutrophil count > 1.40 × 10^9^/L and a platelet count > 150 × 10^9^/L. (6) Subjects must undergo a comprehensive evaluation by a clinician regarding clinical treatment, duration of immunosuppression, and the risk of diseases requiring prevention.

Exclusion Criteria: (1) Individuals who are users of monoclonal antibodies, especially anti-tumor necrosis factor agents. (2) Individuals who are users of intermittent or low-dose chemotherapeutic agents or other immunosuppressive drugs.

### 2.3. Genetic Polymorphism Detection

HLA genotyping was conducted using Sequence-specific primer polymerase chain reaction (PCR-SSP). Specifically, allele-specific primers were designed to amplify the following loci: HLA-A, -B, -Cw, -DRB1, and -DQB1. The amplified products were then compared with standard markers using a scanning imaging analysis system to ascertain the HLA-A, -B, -Cw, -DRB1, and -DQB1 genotypes. Subsequently, the positive products were subjected to gene sequencing.

### 2.4. Statistical Analysis

Statistical analyses were conducted using SPSS 24.0, and graphical visualizations were generated using GraphPad Prism 7.00 and RStudio (version R-4.5.0). The chi-square test was utilized to compare the disparities among children in different response groups with respect to gender, risk groups, transplant status, radiation therapy history, immigration status, and genotype carrier rates. The Shapiro–Wilk test was used to assess the normality of anti-HBs titers across groups, both before and after hepatitis B vaccination. Data that met the normality criteria were subjected to *t*-test analysis. Binary logistic regression was used to evaluate the association between HLA alleles and anti-HBs titers. Statistical significance was defined as a two-tailed *p*-value < 0.05. The *p*-values for differences in HLA gene polymorphisms between the two groups were corrected using the Bonferroni method.

## 3. Results

### 3.1. Analysis of the Immune Response Levels in Children with ALL After Hepatitis B Vaccination

**Figure 1:**
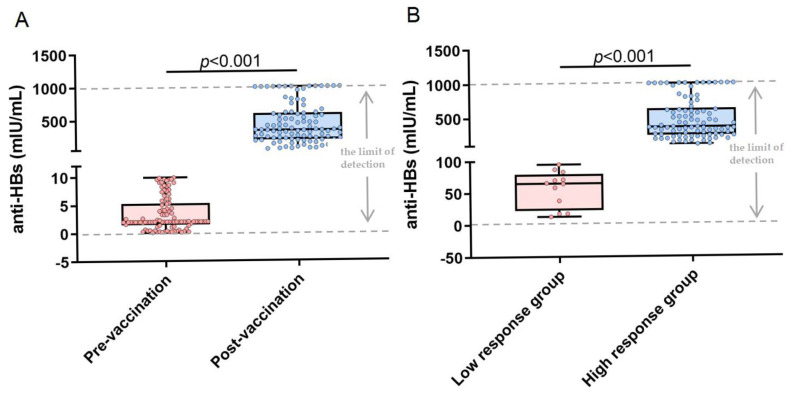
Analysis of anti-HBs titer changes before and after hepatitis B vaccination, and variations between different response groups post-vaccination. (**A**) The average anti-HBs titers in the pre-vaccination and post-vaccination groups were 3.38 ± 2.97 mIU/mL and 429.61 ± 303.13 mIU/mL, respectively. (**B**) The mean anti-HBs titers in the low-response group and high-response group were 56.47 ± 28.38 mIU/mL and 479.93 ± 287.70 mIU/mL, respectively.

Before vaccination, all participants (*n* = 101) were seronegative for anti-HBs, with a mean baseline titer of 3.38 ± 2.97 mIU/mL. After vaccination, a 100% seropositivity rate was achieved, accompanied by significantly elevated anti-HBs titers (429.61 ± 303.13 mIU/mL; *p* < 0.001 compared with baseline) (Figure 1A). We categorized participants into low-responder and high-responder groups based on post-immunization anti-HBs levels exceeding 100 mIU/mL. The results revealed statistically significant differences in antibody levels between the low-response group (56.47 ± 28.38 mIU/mL) and the high-response group (479.93 ± 287.70 mIU/mL; *p* < 0.001) (Figure 1B).

Twelve children (11.88%) in the low-response group and 89 children (88.12%) in the high-response group demonstrated positive anti-HBs titers. No significant inter-group disparities were detected in demographic factors such as age, gender, and immigration status or clinical characteristics including risk groups, transplantation status and radiation therapy history across response categories (all *p* > 0.05) (Table 1).

### 3.2. HLA Genotype Distribution and Immune Response Levels to Hepatitis B Vaccine in Children with ALL

**Figure 2:**
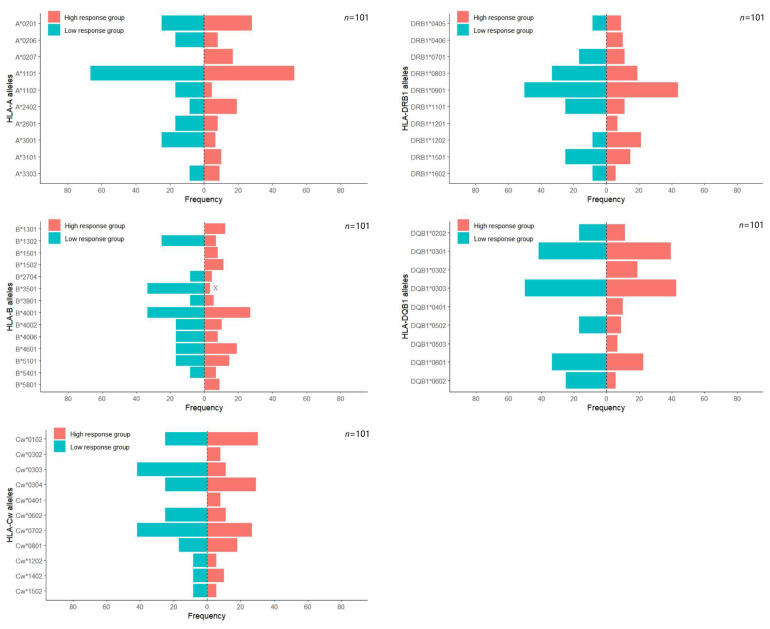
Analysis of frequency differences in HLA-A, B, Cw, DRB1, and DQB1 alleles between different hepatitis B antibody response groups. To reduce statistical bias, all alleles detected in fewer than five samples were excluded. ‘x’ indicates that a significant difference between groups (*p* < 0.00357 for Bonferroni correction). ‘*’ serves as the standard naming symbol to separate locus and allele designations, following the IPD-IMGT/HLA database.

Univariate analysis of HLA allele distribution between vaccine response groups revealed distinct patterns at the HLA-A, -B, -C, -DRB1, and -DQB1 loci. The frequencies of HLA—A alleles were predominantly represented by A*1101 (54.46%), A*0201 (27.72%), A*2402 (17.82%), and A*0207 (14.85%). There were no statistically significant differences in carrier frequencies between the response groups. The major HLA—B alleles were B*4001 (27.72%), B*4601 (18.81%), B*5101 (14.85%), B*1301 (10.89%), and B*4002 (10.89%). Significant inter-group differences in carrier frequency were detected for B*3501 (*p* < 0.01), while no other HLA—B alleles exhibited significant associations. The most prevalent HLA—Cw alleles were Cw*0102 (29.70%), Cw*0304 (28.71%), Cw*0702 (28.71%), Cw*0801 (17.82%), Cw*0303 (14.85%), and Cw*0602 (12.87%). Significant differences in carrier rates were noted for Cw*0303 (*p* < 0.05), whereas other Cw alleles did not differ significantly. The distribution of HLA—DRB1 alleles was characterized by the predominance of DRB1*0901 (44.55%), DRB1*0803 (20.79%), DRB1*1202 (19.80%), DRB1*1501 (15.84%), DRB1*1101 (12.87%), and DRB1*0701 (11.88%). No significant association was observed between any DRB1 allele and vaccine response levels. Likewise, in HLA-DQB1, the most prevalent alleles were DQB1*0303 (43.56%), DQB1*0301 (39.60%), DQB1*0601 (23.76%), DQB1*0302 (16.83%), and DQB1*0202 (11.88%). Similarly, no significant association was found between any DQB1 allele and vaccine response. Since this study involved comparing multiple HLA polymorphic loci between two groups, Bonferroni correction revealed that only the B*3501 carrier frequency showed a significant intergroup difference (*p* < 0.00357 for Bonferroni correction) (Table 2, Figure 2).

### 3.3. Association Between HLA Genotypes and Antibody Levels

We included alleles B*3501, Cw*0303, and DQB1*0602 with *p* < 0.1 from univariate analyses, along with DQB1*0301, which may affect hepatitis B antibody levels, into a binary logistic regression model for in-depth analysis. The findings revealed that B*3501 exerted a statistically significant influence on antibody response levels (OR = 0.113, 95% *CI*: 0.018–0.698, *p* = 0.019), whereas Cw*0303, DQB1*0301, and DQB1*0602 did not (Table 3).

## 4. Discussion

Children who have undergone therapy for ALL, particularly those who received allogeneic hematopoietic stem cell transplantation, experience prolonged immunosuppression and ablation of pre-existing immune memory. Consequently, even those previously vaccinated against hepatitis B may become seronegative for anti-HBs and are at risk for HBV infection post-treatment [3,20]. Revaccination is therefore essential to restore protection during long-term survivorship. This study specifically focuses on ALL survivors who were anti-HBs-negative following completion of therapy, representing a population that requires a complete de novo hepatitis B vaccination series. This study aims to evaluate the immunogenicity of the standard three-dose hepatitis B vaccine regimen in this susceptible population and to investigate the associated HLA genes. Our data indicated that children with ALL who achieve clinical remission exhibit significantly stronger immune responses to the hepatitis B vaccine. This phenomenon likely reflects immunological recovery in stable ALL patients, in whom vaccine responsiveness approaches that of immunocompetent individuals [21]. This finding corroborates the report by Viana et al., which stated that post-chemotherapy booster vaccination significantly elevates anti-HBs titers to protective levels (10 mIU/mL) in ALL patients [21].

This study has not revealed statistically significant differences between the high anti-HBs response group and the low anti-HBs response group in terms of demographic characteristics such as age, gender and immigration status. Furthermore, no significant differences were observed between the two groups in clinical characteristics, including risk groups, transplant status, and radiation therapy history. The findings may have resulted from the fact that all patients included in the present study were from the same specialized hospital, had the same disease type, received similar treatment regimens, and were in a state of near-complete recovery at the time of vaccination, resulting in minimal internal variability. Additionally, with the current sample size, it may be challenging to detect the effects of factors with smaller effect sizes, such as age and gender. Collectively, these factors highlight the role of genetic factors.

Our genetic association study uncovers a remarkable and distinct pattern of HLA-mediated immunity following hepatitis B vaccination. Univariate analysis results suggest that both B*3501 and Cw*0303 may significantly influence antibody levels following hepatitis B vaccination in children with ALL. Notably, compared with the general Han Chinese population (3.0%), the B*3501 allele had a higher carrier frequency (6.93%) among pediatric ALL patients in this study, predominantly observed in the poor-response patient group. No association has been identified linking B*3501 to vaccine-induced HBV antibody levels. Previous research has indicated that the peptide-deficient B*3501 tetramer is capable of directly facilitating the activation of CD8^+^ T cells, which implies that B*3501 exhibits substantial potential for T cell activation [22]. Activated CD8^+^ T cells may enhance the production of anti-HBs after hepatitis B vaccination by assisting the B-cell response, suggesting that B*3501 contributes to the elevation of anti-HBs levels [23].

Nevertheless, the antibody levels generated are insufficient to enable all patients to achieve a complete immune state. Similarly, Cw*0303 were predominantly distributed in the low-response group, which is in line with the study by D. Wolday et al., suggesting that they bind strongly to their respective peptide fragments but exhibit low or no antibody production [24]. Nevertheless, after applying the Bonferroni correction to *p*-values and employing a binary logistic regression model, the association with Cw*0303 weakened and lost statistical independence, whereas that with B*3501 remained robust and significant. These findings indicate that in this study, Cw*0303 may be genetically linked to B*3501; however, its role in anti-HBs production is not entirely independent of B*3501.

Previous studies have demonstrated that specific HLA class II alleles (DRB1 and DQB1) are associated with antibody responses to the hepatitis B vaccine [25]. For example, the alleles DQB1*01, DQB1*0301, DQB1*15, DQB1*0501, DQB1*06, and DQB1*0602 are strongly associated with significantly enhanced antibody responses to hepatitis B vaccines. In contrast, alleles such as DRB1*0301, DRB1*04, DRB1*07, DRB1*1302, and DQB1*02 exhibit the opposite effect. It is noteworthy that in this study, DRB1 and DQB1 did not significantly influence the antibody response levels in children with ALL following hepatitis B vaccine booster administration. This finding suggests that in the pediatric ALL population, the HLA class I pathway, particularly the HLA-B locus, may play an unexpectedly dominant and irreplaceable role in humoral immunity against hepatitis B vaccination.

The study has several limitations. Firstly, the sample size was relatively limited. Secondly, antibody titers were measured over a short-term period, and it was not feasible to evaluate the durability of the vaccine due to the absence of long-term antibody monitoring data in children with ALL. Durability evaluation will be gradually considered in subsequent studies. Finally, this study strictly defined the “no history of hepatitis B infection” group by excluding individuals who were anti-HBc-positive. This implies that our findings cannot be directly generalized to all HBsAg-negative patients, as this population includes individuals with prior infection who retain immune memory and may exhibit stronger vaccine responses. Future studies could compare post-vaccination responses between anti-HBc-negative and anti-HBc-positive patients, and long-term continuous cohort monitoring data may continue to provide supplementary information.

## 5. Conclusions

This study demonstrates that administering three doses of hepatitis B vaccine to children with ALL following recovery is effective, with all recipients exhibiting protective immunity against HBV. HLA-B*3501 may be negatively correlated with hepatitis B vaccine-induced immune response in children with ALL. This finding requires further validation in other cohorts.

## Figures and Tables

**Table 1:** Baseline data analysis of different hepatitis B antibody response groups [$\bar{\chi }$ ± sd/*n* (%)].

Variables		Hepatitis B Antibody Levels After Vaccination		*t*/*χ*^2^	*p * ^a^
		Low Response Group (*n* = 12)	High Response Group (*n* = 89)		
Age (Years)		8.83 ± 2.95	7.60 ± 2.73	1.461	0.147
Gender	Male	5 (4.95)	53 (52.48)	1.383	0.240
	Female	7 (6.93)	36 (35.64)		
Risk groups	Low	7 (6.93)	48 (47.52)	0.218	0.897
	Intermediate	3 (2.97)	28 (27.72)		
	High	2 (1.98)	13 (12.87)		
Transplant status	No	12 (11.88)	77 (76.24)	0.774	0.379
	Yes	0 (0.00)	12 (11.88)		
Radiation therapy history	No	11 (10.89)	78 (77.23)	0.000	1.000
	Yes	1 (0.99)	11 (10.89)		
Immigration status	Migrants	3 (2.97)	33 (32.67)	0.249	0.618
	Residents	9 (8.91)	56 (55.45)		

‘^a^’ indicates the statistical analysis of different hepatitis B antibody response groups.

**Table 2:** Frequency distribution of HLA-A, B, Cw, DRB1 and DQB1 alleles [%].

Alleles	Frequency	Alleles	Frequency	Alleles	Frequency
A*1101	54.46	B*5801	7.92	DRB1*0803	20.79
A*0201	27.72	B*1501	6.93	DRB1*1202	19.80
A*2402	17.82	B*3501	6.93	DRB1*1501	15.84
A*0207	14.85	B*5401	6.93	DRB1*1101	12.87
A*0206	8.91	B*3901	5.94	DRB1*0701	11.88
A*2601	8.91	B*2704	4.95	DRB1*0405	8.91
A*3001	8.91	Cw*0102	29.70	DRB1*0406	8.91
A*3101	8.91	Cw*0304	28.71	DRB1*1201	5.94
A*3303	8.91	Cw*0702	28.71	DRB1*1602	5.94
A*1102	5.94	Cw*0801	17.82	DQB1*0303	43.56
B*4001	27.72	Cw*0303	14.85	DQB1*0301	39.60
B*4601	18.81	Cw*0602	12.87	DQB1*0601	23.76
B*5101	14.85	Cw*1402	9.90	DQB1*0302	16.83
B*1301	10.89	Cw*0302	6.93	DQB1*0202	11.88
B*4002	10.89	Cw*0401	6.93	DQB1*0502	9.90
B*1502	9.90	Cw*1202	5.94	DQB1*0401	8.91
B*1302	8.91	Cw*1502	5.94	DQB1*0602	7.92
B*4006	8.91	DRB1*0901	44.55	DQB1*0503	5.94

‘*’ serves as the standard naming symbol to separate locus and allele designations, following the IPD-IMGT/HLA database.

**Table 3:** Results of binary logistic regression.

Characteristics	*β*	Standard Error	Wald	*p* Value	Odds Ratio	95% *CI*
B*3501	−2.177	0.927	5.511	0.019	0.113	0.018, 0.698
Cw*0303	−0.916	0.851	1.159	0.282	0.400	0.075, 2.121
DQB1*0301	−0.101	0.702	0.021	0.885	0.904	0.228, 3.578
DQB1*0602	−0.939	1.037	0.819	0.365	0.391	0.051, 2.985

Reference group: Individuals not carrying any of the target HLA alleles. ‘*’ serves as the standard naming symbol to separate locus and allele designations, following the IPD-IMGT/HLA database.

## Data Availability

The data presented in this study are available on request from the corresponding author, given that the data involves the personal privacy of research subjects.

## Abbreviations

The following abbreviations are used in this manuscript:

HLA	Human leukocyte antigen
ALL	Acute lymphoblastic leukemia
Anti-HBs	Hepatitis B surface antibody
PCR-SSP	Sequence-specific primer polymerase chain reaction
DALYs	Disability-adjusted life years
HBV	Hepatitis B virus
MHC	Major histocompatibility complex
DNA	Genomic deoxyribonucleic acid
HBsAg	Hepatitis B surface antigen
Anti-HBc	Hepatitis B core antibody

## References

[1] Wang L., Yao X., Yang L. (2025). Global, regional, and national burden of children and adolescents with acute lymphoblastic leukemia from 1990 to 2021: A systematic analysis for the global burden of disease study 2021. Front. Public Health.

[2] Rajendran P.V., Thankamony P., Rajeswari B., Sojamani G.C., Nair M., Parukuttyamma K., Krishna Km J. (2023). Loss of protective anti-HBs titers and seroconversion to hepatitis B vaccination in children during chemotherapy for acute lymphoblastic leukemia. Pediatr. Blood Cancer.

[3] Xiong D., Cai W., Zhao W. (2024). Risk factors of HBV reactivation in leukemia patients with resolved HBV infection after allogeneic hematopoietic stem cell transplantation. Clin. Res. Hepatol. Gastroenterol..

[4] Pattyn J., Hendrickx G., Vorsters A., Van Damme P. (2021). Hepatitis B Vaccines. J. Infect. Dis..

[5] Sarfaraz N., Somarowthu S., Bouchard M.J. (2023). The interplay of long noncoding RNAs and hepatitis B virus. J. Med. Virol..

[6] Yan R., Sun M., Yang H., Du S., Sun L., Mao Y. (2025). 2024 latest report on hepatitis B virus epidemiology in China: Current status, changing trajectory, and challenges. Hepatobiliary Surg. Nutr..

[7] Han J.W., Yang H., Lee H.L., Bae S.H., Choi J.Y., Lee J.W., Kim H.J., Lee S., Cho S.G., Min C.K. (2016). Risk factors and outcomes of hepatitis B virus reactivation in hepatitis B surface antigen negative patients with hematological malignancies. Hepatol. Res..

[8] Guruprasad B., Kavitha S., Aruna Kumari B.S., Vijaykumar B.R., Sumati B.G., Mahua S., Appaji L., Jayshree R.S. (2014). Risk of hepatitis B infection in pediatric acute lymphoblastic leukemia in a tertiary care center from South India. Pediatr. Blood Cancer.

[9] Mahmood F., Xu R., Awan M.U.N., Song Y., Han Q., Xia X., Wei J., Xu J., Peng J., Zhang J. (2023). HBV Vaccines: Advances and Development. Vaccines.

[10] Tahir A., Shinkafi S.H., Alshrari A.S., Yunusa A., Umar M.T., Hudu S.A., Jimoh A.O. (2024). A Comprehensive Review of Hepatitis B Vaccine Nonresponse and Associated Risk Factors. Vaccines.

[11] Bello N., Hudu S.A., Alshrari A.S., Imam M.U., Jimoh A.O. (2024). Overview of Hepatitis B Vaccine Non-Response and Associated B Cell Amnesia: A Scoping Review. Pathogens.

[12] Liu D.H., Mou F.F., An M., Xia P. (2023). Human leukocyte antigen and tumor immunotherapy (Review). Int. J. Oncol..

[13] Qiu J., Zhang S., Feng Y., Su X., Cai J., Chen S., Liu J., Huang S., Huang H., Zhu S. (2024). Efficacy and safety of hepatitis B vaccine: An umbrella review of meta-analyses. Expert Rev. Vaccines.

[14] Li X., Zhou Q., Lu Z., Huang R., Lin D., Xu J., Yu X., Li X. (2024). Association of HLA-DRB1 alleles with status of antibodies to hepatitis B surface and e antigen. J. Med. Virol..

[15] Naderi M., Hosseini S.M., Behnampour N., Besharat S., Shahramian I., Khoshnia M., Moradi A. (2024). Host and Viral Factors Influencing Chronic Hepatitis B Infection Across Three Generations in a Family. Curr. Microbiol..

[16] Mentzer A.J., Dilthey A.T., Pollard M., Gurdasani D., Karakoc E., Carstensen T., Muhwezi A., Cutland C., Diarra A., da Silva Antunes R. (2024). High-resolution African HLA resource uncovers HLA-DRB1 expression effects underlying vaccine response. Nat. Med..

[17] Chinese Prevention Medicine Association, National Immunization Program, Chinese Center for Disease Control and Prevention (2012). Technical guide for adult hepatitis B immunization in China. Chin. J. Viral. Dis..

[18] Public Health England (2025). Hepatitis B. Green Book: Immunisation Against Infectious Disease.

[19] Baruti K., Lentz K., Anderson M., Ajibola G., Phinius B.B., Choga W.T., Mbangiwa T., Powis K.M., Sebunya T., Blackard J.T. (2020). Hepatitis B virus prevalence and vaccine antibody titers in children HIV exposed but uninfected in Botswana. PLoS ONE.

[20] de Koning C., Plantinga M., Besseling P., Boelens J.J., Nierkens S. (2016). Immune Reconstitution after Allogeneic Hematopoietic Cell Transplantation in Children. Biol. Blood Marrow Transpl..

[21] Viana S.S., Araujo G.S., Faro G.B., da Cruz-Silva L.L., Araújo-Melo C.A., Cipolotti R. (2012). Antibody responses to Hepatitis B and measles-mumps-rubella vaccines in children who received chemotherapy for acute lymphoblastic leukemia. Rev. Bras. Hematol. Hemoter..

[22] Geng J., Altman J.D., Krishnakumar S., Raghavan M. (2018). Empty conformers of HLA-B preferentially bind CD8 and regulate CD8(+) T cell function. eLife.

[23] Peng J., Yao X., Yuan C., Liu X., Xia R., He J., Li R., Yao Y. (2022). The Investigation of Hepatitis B Vaccine Immune Responses in Occult Hepatitis B Virus-Infected Patients. Front. Immunol..

[24] Wolday D., Fung C.Y.J., Morgan G., Casalino S., Frangione E., Taher J., Lerner-Ellis J.P. (2023). HLA Variation and SARS-CoV-2 Specific Antibody Response. Viruses.

[25] Li Z.K., Nie J.J., Li J., Zhuang H. (2013). The effect of HLA on immunological response to hepatitis B vaccine in healthy people: A meta-analysis. Vaccine.

